# Reduction of the hand representation in the ipsilateral primary motor cortex following unilateral section of the corticospinal tract at cervical level in monkeys

**DOI:** 10.1186/1471-2202-6-56

**Published:** 2005-08-31

**Authors:** Eric Schmidlin, Thierry Wannier, Jocelyne Bloch, Abderraouf Belhaj-Saif, Alexander F Wyss, Eric M Rouiller

**Affiliations:** 1 Unit of Physiology and Program in Neurosciences, Department of Medicine, Faculty of Sciences, University of Fribourg, Chemin du Musée 5, CH-1700 Fribourg, Switzerland; 2 Brain Research Institute, Department of Neuromorphology, University and ETH Zurich, Winterthurerstrasse 190, CH-8057 Zürich, Switzerland; 3 Department of Neurosurgery, Neurosurgery Clinic, University Hospital of Lausanne, Rue du Bugnon, CH-1011 Lausanne, Switzerland

## Abstract

**Background:**

After sub-total hemi-section of cervical cord at level C7/C8 in monkeys, the ipsilesional hand exhibited a paralysis for a couple of weeks, followed by incomplete recovery of manual dexterity, reaching a plateau after 40–50 days. Recently, we demonstrated that the level of the plateau was related to the size of the lesion and that progressive plastic changes of the motor map in the contralesional motor cortex, particularly the hand representation, took place following a comparable time course. The goal of the present study was to assess, in three macaque monkeys, whether the hand representation in the ipsilesional primary motor cortex (M1) was also affected by the cervical hemi-section.

**Results:**

Unexpectedly, based on the minor contribution of the ipsilesional hemisphere to the transected corticospinal (CS) tract, a considerable reduction of the hand representation was also observed in the ipsilesional M1. Mapping control experiments ruled out the possibility that changes of motor maps are due to variability of the intracortical microstimulation mapping technique. The extent of the size reduction of the hand area was nearly as large as in the contralesional hemisphere in two of the three monkeys. In the third monkey, it represented a reduction by a factor of half the change observed in the contralesional hemisphere. Although the hand representation was modified in the ipsilesional hemisphere, such changes were not correlated with a contribution of this hemisphere to the incomplete recovery of the manual dexterity for the hand affected by the lesion, as demonstrated by reversible inactivation experiments (in contrast to the contralesional hemisphere). Moreover, despite the size reduction of M1 hand area in the ipsilesional hemisphere, no deficit of manual dexterity for the hand opposite to the cervical hemi-section was detected.

**Conclusion:**

After cervical hemi-section, the ipsilesional motor cortex exhibited substantial reduction of the hand representation, whose extent did not match the small number of axotomized CS neurons. We hypothesized that the paradoxical reduction of hand representation in the ipsilesional hemisphere is secondary to the changes taking place in the contralesional hemisphere, possibly corresponding to postural adjustments and/or re-establishing a balance between the two hemispheres.

## Background

Although voluntary dexterous movements of the hand are mainly under control of motor cortical areas in the opposite hemisphere, there is evidence that the ipsilateral motor cortex may also contribute, but to a lesser extent. For instance, the activity of single neurons in the primary (M1), supplementary (SMA), premotor (PM) and cingulate (CMA) motor cortical areas was found to be modulated when monkeys performed movements with the ipsilateral hand. [1-7]. Intracortical microstimulations in the primary motor cortex in monkeys were reported to evoke not only the expected movements of the contralateral digits [8] but also responses of the ipsilateral fingers [9]. In human subjects, there is increasing evidence that the motor cortex is involved in the control of ipsilateral hand movements (e.g. [10-16]). The possible contribution of the motor cortex in the control of the ipsilateral hand may be important for normal function, although its precise role has not been elucidated yet. Furthermore, it has been anticipated that the ipsilateral motor cortex may be crucial for recovery of motor function of a paretic hand after unilateral brain lesion, such as after stroke (e.g. [17-20]). However, the involvement of the intact hemisphere in the control of the ipsilateral paretic hand remains a matter for debate. [21-23]. Moreover, there is clear evidence, in both monkeys and human subjects, that a significant re-organization of motor maps takes place in the affected hemisphere after unilateral lesion of M1. [24-28], a re-arrangement crucially involved in the functional recovery of the paretic hand [25].

Regarding lesions of the spinal cord, several studies reported plastic changes of motor maps in the cerebral cortex in both monkeys [29-31] and human subjects [32-37]. In a recent report, we described in detail the changes of motor maps that occurred in the motor cortex contralateral to a unilateral section of the corticospinal (CS) tract at cervical level C7/C8 [31]. Several months after the lesion, the contralesional hemisphere showed a dramatic decrease of the hand representation, compared with before the lesion, ranging from 69 to 97 % depending on the site and extent of the lesion. The progressive changes in hand representation occurred during the 30–40 days post-lesion, in parallel to the functional recovery of the affected hand. Finally, we demonstrated that the re-arranged contralesional motor cortex with a diminished hand representation was crucial for the functional recovery of the affected hand, since its reversible inactivation abolished the recovered motor performance [31]. The goal of the present report was to address the issue of possible changes in the hemisphere ipsilateral to the unilateral cervical lesion, in other words the ipsilesional hemisphere. More specifically, the following questions were addressed:

- Does a unilateral section of the CS tract at cervical level affect the hand representation in the ipsilesional motor cortex? If yes, to what extent as compared to the contralesional hemisphere? Based on the small proportion of undecussated CS axons (about 5–10%; [38-40]), one would predict a very limited, if any, impact of a unilateral cervical lesion on the ipsilesional motor cortex.

- In the context of the recovery of motor control from unilateral section of the CS tract, does the ipsilesional hemisphere play a role, in addition to the substantial contribution of the contralesional hemisphere?

## Results

### Unilateral section of the CS tract at cervical level

**Figure 1:**
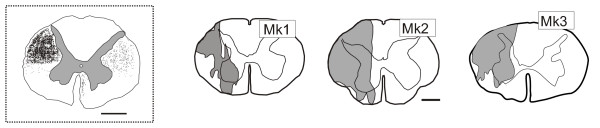
**Location and extent of unilateral cervical lesion in Mks 1–3. **Location and extent of the unilateral lesion (grey area), performed at C7/C8 level in Mk1, Mk2 and Mk3, shown on a reconstructed frontal view of cervical cord (three rightmost panels). The inset on the left is a frontal section of the macaque monkey cervical cord showing the distribution of CS axons (black dots) as a result of injection of the anterograde tracer Biotinylated Dextran Amine (BDA) in the right motor cortex (as recently reported in [41]). The CS axons originating from the right hemisphere are distributed mainly in the left dorsolateral funiculus (90–95%), representing the decussated CS tract. The uncrossed CS tract (5–10%) comprises, on the right side, axons in the dorsolateral funiculus and in the ventral funiculus. Scale bars = 2 mm.

Three monkeys (Mk1, Mk2 and Mk3) were subjected to a unilateral section of the CS tract at cervical level. The extent and precise position of the lesion were assessed from reconstructions of cervical cord histological sections cut in the paralongitudinal plane as explained previously [31,41]. The lesion was finally represented for Mks1-3 on a transverse reconstruction of the cervical cord (Fig. 1). For comparison, the left inset of Figure 1 shows the distribution of the CS axons at cervical level after injection of the anterograde tracer Biotinylated Dextran Amine (BDA) in the right motor cortex, as recently described [41]. The smallest lesion was in Mk1 (Fig. 1), which however interrupted most of the dorsolateral funiculus comprising the crossed CS axons originating from the contralesional hemisphere. A restricted part of the CS axons located in the most ventral and lateral part of the dorsolateral funiculus (about 5%) may have however been preserved in Mk1. In contrast, the lesion was larger in Mk2 and Mk3, for which the left dorsolateral funiculus was, respectively, completely or nearly completely transected (Fig. 1). Overall, as intended, the lesion affected substantially if not totally the left dorsolateral funiculus in Mks1-3, above the spinal segments where the motoneurons controlling hand muscles are located [42]. Regarding the uncrossed CS tracts originating from the ipsilesional hemisphere, the comparison with the inset of Figure 1 indicates that the lesion in Mks1-3 interrupted most (Mk1) or all (Mks2-3) of the uncrossed CS axons located in the dorsolateral funiculus, but not the few CS axons running in the ventral funiculus.

### Mapping of ipsilesional M1 hand area before and after cervical cord lesion: ICMS data

**Figure 2:**
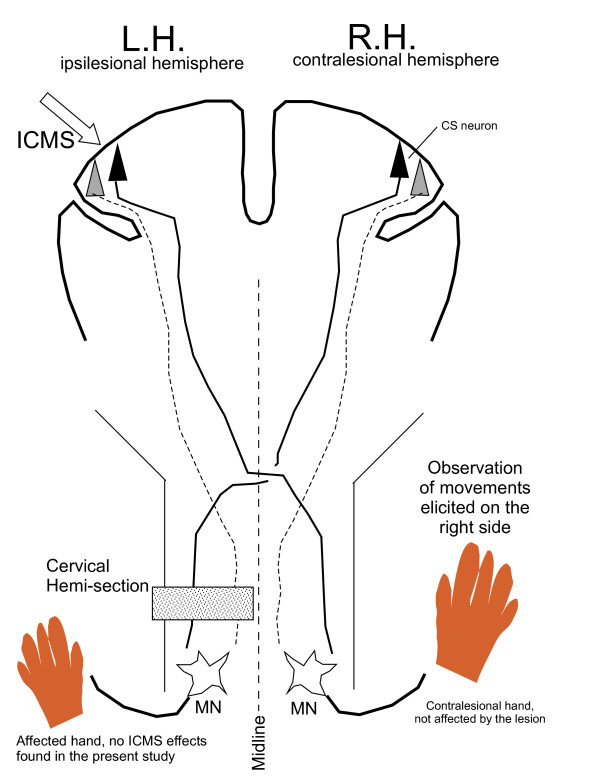
**Experimental paradigm in Mks1-3. **A cervical (left) sub-hemi-section at C7/C8 interrupted the main crossed corticospinal tract originating from the contralesional (right) hemisphere (RH) and the small, uncrossed, corticospinal tract, originating from the ipsilesional (left) hemisphere (LH). In order to assess the functional changes induced by the lesion in the ipsilesional hemisphere, electrode penetrations were performed in order to deliver intracortical microstimulations (ICMS) in the ipsilesional hemisphere. The effects of the ICMS were observed on the contralesional (right) side of the body, with emphasis on the contralesional hand, not affected by the lesion. In the present study, no effects were observed in the ipsilesional hand, affected by the lesion, for ICMS applied to the ipsilesional hemisphere. In other words, all ICMS effects reported in the present study are for the contralesional hand. MN = motoneuron

The experimental paradigm is depicted in Figure 2. As a result of the left cervical lesion, the ipsilesional hand was paralyzed, but showed incomplete recovery in about 30–40 days [31]. In the present report, the ipsilesional hemisphere was investigated electrophysiologically, using intracortical microstimulation (ICMS) at two time points: before the lesion (pre-lesion motor map) and a few months after the lesion (post-lesion motor map) when the monkey has reached a plateau of manual dexterity recovery. The pre- and post-lesion cortical maps established in the ipsilesional hemisphere report on the movement elicited by ICMS on the contralesional (right) body side, with a focus on the hand not affected by the lesion (Fig. 2). In the present experiments, as a result of ICMS in the ipsilesional hemisphere, no movements were observed for the ipsilesional hand.

**Figure 3:**
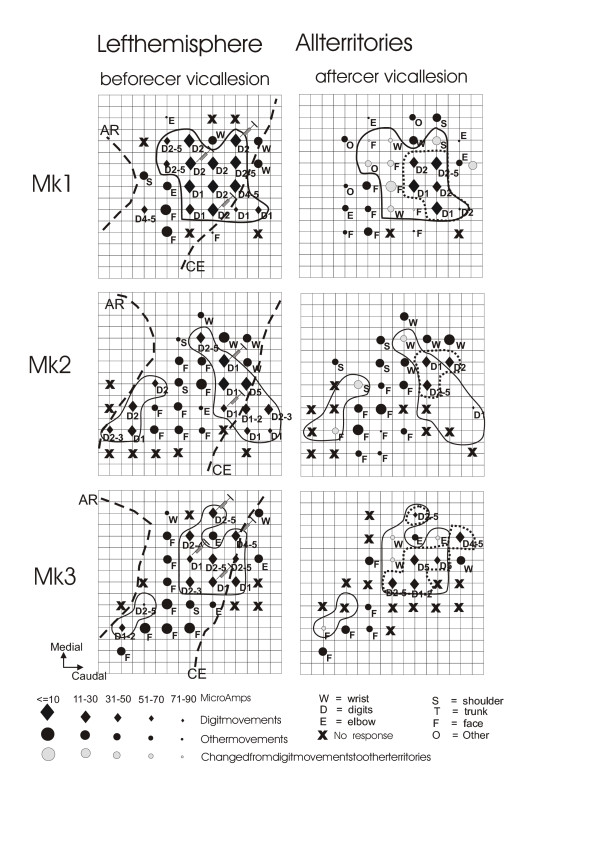
**Intracortical stimulation mapping of the ipsilesional motor cortex. **Somatotopic map in the motor cortex on the left (ipsilesional) hemisphere in the region of the hand area, before (left column) and after (right column) the cervical lesion for Mk1, Mk2 and Mk3 (from top to bottom). The data are presented on a surface view of the cerebral cortex, the portion accessible via the chronic recording chamber. The pre-lesion maps were established by daily ICMS sessions conducted during the 3 months preceding the lesion. The post-lesion maps were derived from daily ICMS sessions starting 2, 4.5 and 5 months after the lesion in Mk1, Mk2 and Mk3, respectively and lasting about 2 months each. Each symbol represents the location on the cortical surface of the penetration with an electrode for ICMS. The ICMS data given for each symbol is representative for the site of stimulation where the effect was observed at the lowest current intensity along the considered electrode track. The letter next to each symbol indicates the body territory activated at threshold for each track (see letter codes in the bottom right). The size of the symbols indicates the current intensity at threshold (in μA; see bottom left). Hand area(s), outlined by a solid line, was defined as a cortical region pre-lesion where ICMS at lowest threshold elicits movements of the fingers (electrode tracks represented by diamonds). Symbol X means that ICMS did not elicit any visible movement of muscles. The pointed lines outline the hand area, as defined post-lesion. Symbols in grey are for sites belonging to the hand area before lesion, which became part of other territories post-lesion. The grid in the background indicates steps of 1 mm. Syringes point to sites where muscimol was infused in order to inactivate M1 (see text). In order to make sure that the entire "post-lesion" hand representation was reversibly inactivated, the sites of infusion of muscimol were selected based on the "pre-lesion" map, exhibiting a larger hand representation than post-lesion. On the pre-lesion maps (left column), the approximate positions of the central (CE) and arcuate (AR) sulci are indicated by dashed lines.

The motor cortical map established before the cervical lesion (left column in Fig. 3) exhibits the presence of a clear hand area, defined here as the outline of penetration points represented on the surface along which the lowest threshold ICMS effect was a movement of digits (D1- D5 symbols) of the opposite (right) hand. In the three monkeys, the hand area in M1 is surrounded by points where ICMS elicited movements of other body territories, such as face (F), wrist (W), elbow (E) or shoulder (S). In Mk2 and Mk3, a slightly more rostral position of the chronic recording chamber allowed us to investigate more anterior regions of the motor cortex. There we found a second, smaller hand area, characterized by slightly higher thresholds to elicit hand movements than the main hand area in M1 (Fig. 3, left panel of middle and bottom rows). To address the question of whether such somatotopic representations are influenced in the ipsilesional motor cortex as a result of the unilateral cervical lesion (as recently reported for the contralesional hemisphere, see [31]), the ipsilesional hemisphere was re-mapped a few months post-lesion (Fig. 3, right column). Surprisingly, there were substantial changes in motor map in the ipsilesional hemisphere as well. The area of the hand representation in M1 projected on the surface of the hemisphere was reduced by a factor of 52% in Mk1, 77% in Mk2 and 43% in Mk3 (Fig. 3). Clearly, some ICMS sites which elicited hand movements before the lesion were replaced at threshold by effects on other body territories post-lesion (gray symbols in Fig. 3) or became non microexcitable (X symbols within the outlined area in the right column of Fig. 3).

**Figure 4:**
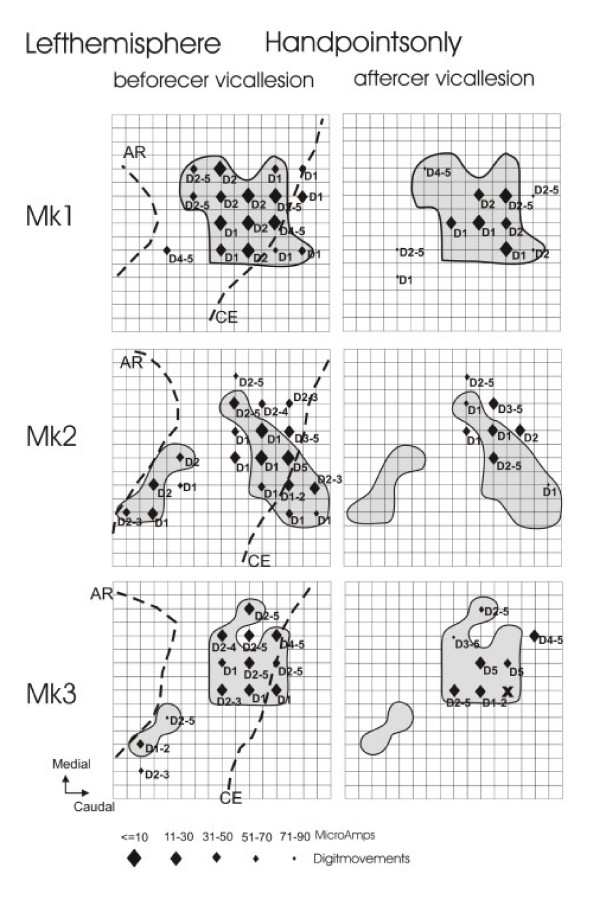
**Hand representation in the ipsilesional motor cortex. **Somatotopic map in the ipsilesional hemisphere as in Fig. 3, but represented are only the electrode penetrations in which digit movements were observed. The gray zones correspond to the hand areas as defined in Figure 3. Outside these areas, other electrode tracks are represented, along which ICMS produced digit movements, but at an intensity that was higher than effects observed for other body territories. Same conventions as in Figure 3.

The ICMS maps shown in Figure 3 display only one point on the surface of the motor cortex for each electrode penetration and only the effect of the lowest threshold was indicated. As a result, other ICMS sites where digit movements were present (but for an intensity of stimulation higher than the lowest threshold for the corresponding electrode track) do not appear. For this reason, all electrode penetrations where digit movements have been observed (irrespective of the intensity of stimulation) are represented in Figure 4, in which only the ICMS data pertaining to digit movements are represented. As expected, the hand territories in Figure 4 (left column) spread on a larger zone of the motor cortex than in the map considering only ICMS effects at threshold (Fig. 3). Nevertheless, as a result of the unilateral cervical lesion, some of these additional hand ICMS sites also disappeared or a few of them exhibited a higher threshold after lesion as compared to the pre-lesion map (Fig. 4).

Instead of considering only one ICMS site (at lowest threshold) per electrode penetration as in Figure 3, or only one body territory (the hand) as in Figure 4 (also with only one ICMS site representing a given electrode penetration), one can also conduct an analysis taking into consideration all ICMS sites tested, pre- and post-lesion. In this way, one avoids (as in Figures 3 and 4) the underestimation of the hand territory lying in the rostral bank of the central sulcus, where a penetration all the way down the bank was represented by a single point on the surface map. In the post-lesion ICMS sessions, most electrode penetrations that were performed during the pre-lesion ICMS sessions were repeated. Table 1 lists the distribution, in absolute numbers as well as in percentage, of all ICMS sites tested in the three lesioned monkeys, separately for the pre- and post-lesion ICMS sessions. The total numbers of ICMS sites (rightmost column in Table 1) indicate that roughly comparable numbers of stimulation sites were tested pre- and post-lesion. For each monkey, the ICMS data points were divided into three groups based on the territories activated or absence of effect: i) digit movements (hand territory), ii) movements of "other" body territories (mainly wrist, elbow, shoulder or face), iii) ICMS sites which did not elicit any visible movement (corresponding to non-microexcitable sites). In all three lesioned monkeys, the comparison pre- and post-lesion of the distribution of ICMS effects observed at all sites tested clearly shows a decrease of the number of ICMS sites eliciting digit movements as a result of the lesion. In percentages, the number of digit ICMS sites was two to nearly three times lower post- than pre-lesion across monkeys, in line with the decrease of the surface of the hand area seen in Figures 3 and 4. The digit ICMS sites lost as a result of the lesion were replaced in most cases by non-microexcitable sites, as shown by the substantial increase (by a factor of 1.3 – 1.5 across monkeys) of such points comparing pre- and post-lesion data (Table 1). For the other body territories, the number of sites slightly increased post-lesion in Mk1 and Mk2 (suggesting replacement of a few original digit points), but decreased in Mk3. As shown in Figure 3, ICMS penetrations that belonged to the hand area before lesion (where ICMS elicited digit movements at lowest threshold) were replaced post-lesion mainly by penetrations which became part of the wrist, elbow, shoulder or even face representations.

### Comparison of ICMS thresholds

Although both the surface of the hand representation and the number of digit sites decreased in the contralesional M1, it was observed that the ICMS thresholds in the hand area post-lesion were not significantly higher than the ICMS thresholds derived from the hand area pre-lesion [31]. This analysis demonstrated that, at least for the contralesional hemisphere, the hand area though decreased in size as a result of the unilateral cervical lesion did not change with respect to its excitability to address the motoneurons of hand muscles, as well as the muscles of other body territories (face, wrist, elbow, shoulder, trunk). The question here is whether the ICMS thresholds were also kept unchanged pre- versus post-lesion in the ipsilesional hemisphere? To address this question, we compared the ICMS thresholds in the ipsilesional hemisphere required to elicit movements from stimulation at the same stereotaxic points before and after the lesion, in the latter case when the manual dexterity score reached the plateau. In contrast to Figure 3 where only the best ICMS site along each electrode penetration was represented, all ICMS sites of stimulation were considered. In the three monkeys, there was no systematic and statistically significant difference between the ICMS thresholds obtained in the ipsilesional hemisphere, before and after the unilateral cervical cord lesion, neither for the hand nor for other body territories (wrist, elbow, shoulder and face).

### Variability of the ICMS mapping method

**Figure 5:**
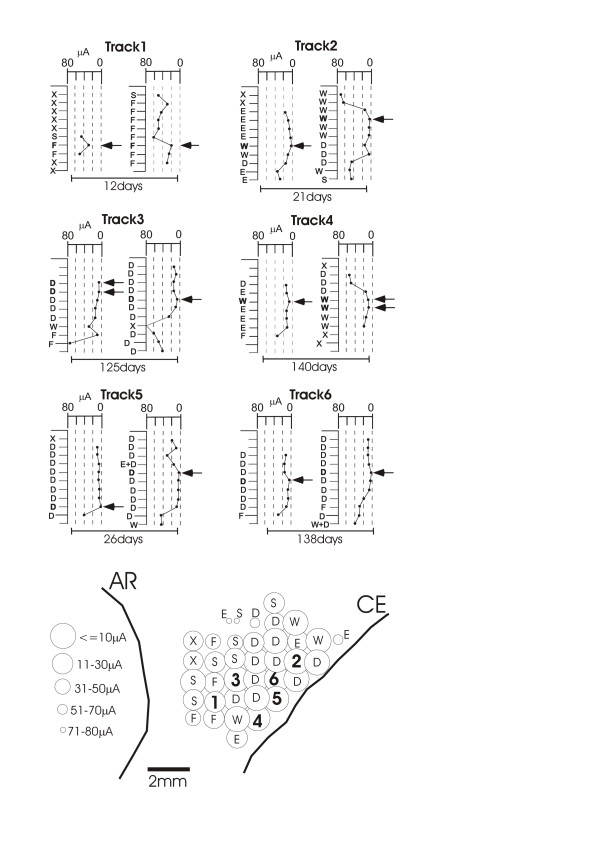
**Reproducibility of the intracortical microstimulation method. **To investigate the variability of the method, six ICMS electrode tracks (1 to 6) were each performed at two time points, separated by a time interval indicated in days (12, 21, 125, 140, 26, 138) below the horizontal bar. For each track, the penetration drawn on the left is the first one whereas the second, performed at the same location, is displayed on the right. For each penetration, the tics along the vertical line represent the sites of stimulation, at a distance of 1 mm. Usually, the first stimulation site is located 2 mm below the surface of the dura. The horizontal scale (in μAmps) indicates for each stimulation site the lowest current at which the effect was observed. The body territory activated by the ICMS is indicated as follows: "D" = digit; "E" = elbow; "F" = face; "S" = shoulder; "W" = wrist; "X" = non-microexcitable site. Along each penetration, the arrows (and bold letters) indicate the ICMS threshold for the entire penetration (criterion taken to define the body territory for the corresponding location on the surface map). The bottom panel is a surface representation of the left hemisphere of the intact Mk4, in which the six electrode penetrations represented above were performed twice (indicated by the numbers 1–6). The other circles with the same letter code as above represent the other electrode penetrations performed in the left hemisphere of Mk4. The size of the circles represents the ICMS threshold obtained for the corresponding electrode penetration. CE = central sulcus; AR = arcuate sulcus; rostral is to the left and medial towards top.

One may argue that the size reduction of the hand representation post-lesion, as compared to the pre-lesion situation (Figs. 3 and 4), is due to the intrinsic variability of the ICMS method. In other words, what is the variability of ICMS sites elicited along two electrode penetrations performed at two distinct time points in an intact monkey? This question was addressed in an intact monkey (Mk4), in which six electrode penetrations taken from the left hemisphere were repeated at time intervals ranging between 12 and 140 days (Fig. 5). For instance, three electrode penetrations located in the hand area (tracks 3, 5 and 6), in which movements of the digits were observed at the lowest threshold site when the electrode was inserted the first time (arrows in the left columns), exhibited again a movement of the digits at the lowest effective current intensity 125, 26 and 138 days later. Interestingly, note that the ICMS thresholds were highly comparable at the two time points (Fig. 5). Along these three electrode tracks (3, 5 and 6), the sequence of territories activated at the consecutive ICMS sites (with a step of 1 mm along the penetration) remained generally comparable at the two time points.

Two other electrode penetrations were taken from the wrist representation (tracks 2 and 4) and repeated at two time points, separated by an interval of 21 and 140 days, respectively (Fig. 5). In both tracks, the first penetration yielded several ICMS sites at which the elbow ("E") articulation was activated, replaced in the second penetrations by wrist movements in most cases. However, the lowest efficient current intensity corresponded to a wrist ("W") movement in the two tracks, both during the first and the second penetrations (Fig. 5). Again, as for digits territories, the threshold obtained for these two wrist territories remained similar at the two time points tested. Along the same line, in the electrode track located in the face representation (track 1), at the two time points tested (12 days apart), ICMS at threshold elicited movements of face muscles (Fig. 5). In summary, from the six electrode tracks repeated at two time points (Fig. 5), one can conclude that, in spite of some variability at some ICMS sites, the territory assigned to each track as defined by the effect observed at threshold did not change, even when the time interval was as long as 140 days. These observations support the notion that the size reduction of the hand area observed here in Figures 3 and 4 cannot be explained by the intrinsic variability of the ICMS method and thus are indeed related to the cervical lesion. Along the same line, a few individual electrode penetrations repeated twice before the cervical lesion in Mks1-3 also showed reproducibility of motor map body territories assessed by ICMS [31].

### Does the ipsilesional (reduced in size) hand area contribute to the post-lesion recovery of the affected hand?

**Figure 6:**
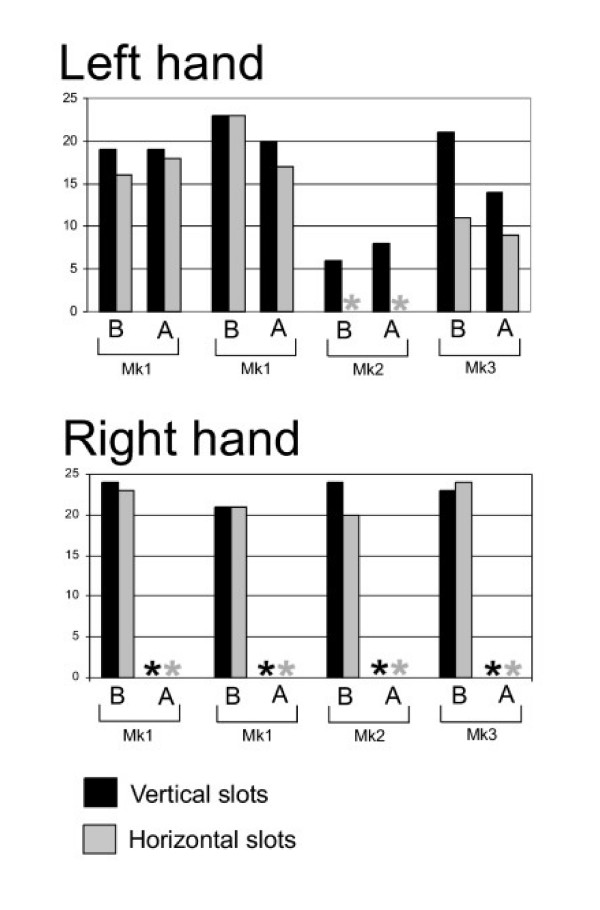
**Effect of reversible inactivation of the ipsilesional motor cortex on manual dexterity. **Effects of reversible inactivation, by infusion of muscimol, of the ipsilesional M1 hand area on the manual dexterity scores for the ipsilesional (left) hand and the contralesional (right) hand. The manual dexterity scores (ordinate) are the number of pellets successively grasped using the precision grip (opposition of thumb and index finger) in the modified Brinkman board task in 45 sec. All inactivation sessions took place post-lesion at time points indicated in the method section. The manual dexterity score was established before ("B") and after ("A") the infusion of muscimol, separately for the vertical and horizontal wells. Two inactivation sessions were conducted in Mk1 and only one in the two other monkeys. The stars are for scores equal to zero, in other words when muscimol infusion completely abolished the corresponding performance. The drop of all scores to zero after infusion ("A" bars) for the right hand demonstrates the efficacy of the inactivation. See text for more detailed description of the results. The star in the session "before muscimol" for Mk2 indicates that this animal did not recover any ability to grasp pellets in the horizontal slots.

To address this question, reversible inactivation sessions of M1 in the ipsilesional hemisphere were performed between 3 to 5 months post-lesion, when the manual dexterity score derived from the Brinkman board test had reached a plateau, indicative of the maximal level of recovery. In a typical inactivation session obtained by infusion of muscimol, the manual dexterity task was initially performed just before the muscimol infusion and repeated 40 – 45 minutes afterwards- a time point at which its effect is well established. A pre- and post-inactivation manual dexterity score was thus determined for each hand, corresponding to the bars "B" (= before) and "A" (= after) in Figure 6. Before infusion of muscimol ("B"), the manual dexterity score of the ipsilesional hand was that corresponding to the plateau of incomplete recovery (Fig. 6, top panel). In the three lesioned monkeys, a reversible inactivation of the ipsilesional M1 did not noticeably affect the recovered manual dexterity score of the ipsilesional hand (bars under "B" and "A" in the top panel of Fig. 6 are comparable for each animal). In other words, the level of the recovery plateau for the ipsilesional hand was not modified by inactivation of the ipsilesional M1 hand area. In sharp contrast, as recently reported [31], a reversible inactivation of the contralesional M1 led to a complete loss of the recovered performance of the ipsilesional hand (not shown here). The data presented in the bottom panel of Figure 6 for the contralesional hand demonstrate the efficacy of the muscimol reversible inactivation method since, as expected, the pharmacological lesion of the left M1 hand area dramatically suppressed the ability of the contralesional hand to perform the precision grip. Recovery from inactivation using muscimol is slow (several hours) and therefore could not be tested within the same inactivation session. However, the day after, the animal had fully recovered its manual dexterity from before the inactivation session.

### Does the size reduction of the ipsilesional M1 hand area lead to a post-lesion motor deficit of the hand contralateral to the cervical hemi-section?

**Figure 7:**
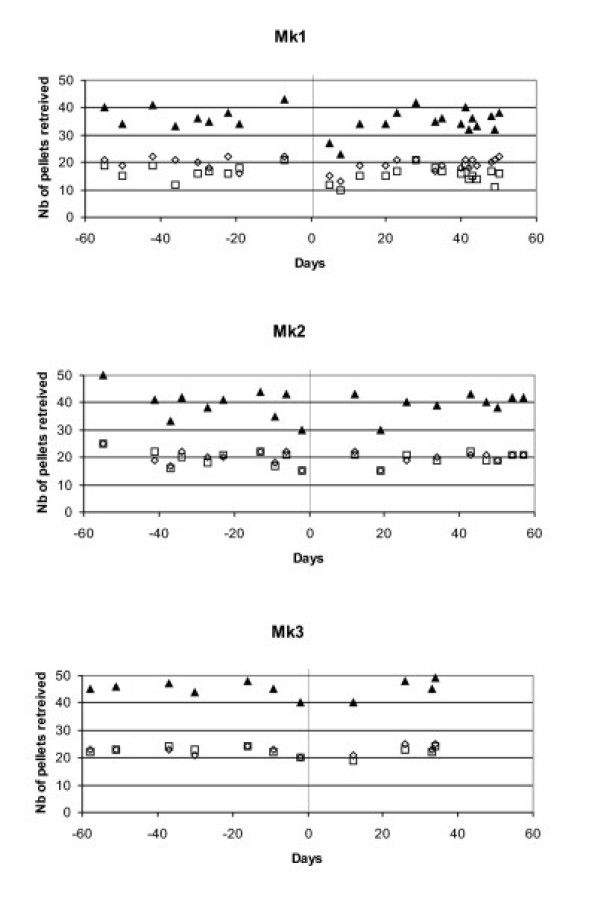
**Dexterity of the contralesional hand. **Manual dexterity of the contralesional hand, assessed with the Brinkman-board task and given by the number of pellets retrieved during 45 sec. (ordinate) as a function of time (abscissa, day of the corresponding session). For each monkey, pre-lesion sessions were performed during the 2 months preceding the lesion (day 0; vertical line), as well as 2 months post-lesion. Squares are for the number of pellets retrieved from the horizontal slots and diamonds from the vertical slots. The triangles are for the sum of the horizontal and vertical slots.

The manual dexterity of the contralesional hand was assessed during several weeks pre- and post-lesion using the "Brinkman board" task (Fig. 7). These data can determine whether a substantial reduction of the size of the M1 ipsilesional hand area is accompanied by a decrease of the manual dexterity of the contralesional hand. As shown by the behavioral plots, the manual dexterity scores for the three lesioned monkeys are very comparable pre- and post-lesion (Fig. 7). The manual dexterity of the contralesional hand is thus not affected by the unilateral cervical lesion or by the plastic changes taking place in the ipsilesional motor cortex, as measured by the Brinkman board test. In Mk1, there was a slight decrease of the manual dexterity score for a few days following the lesion, before returning to the pre-lesion level. Such a transient effect might reflect a modification of the posture of the animal following the cervical lesion, rather than a direct effect on the contralesional hand.

To further test the motor skill of the forelimbs of Mks 1–3, the three lesioned animals also performed the so-called "reach and grasp drawer" task (see methods). In addition to precision grip skill, the drawer task tests the ability of the monkey to develop force with one or the other forelimb. The analysis of these data demonstrated that there was a deficit (time intervals and their variability were increased) for the ipsilesional arm but not for the contralesional forelimb (not shown). In line with the "Brinkman board" task, the drawer task did not show any deficit of the contralesional hand in relation to the size reduction of the ipsilesional M1 hand area.

## Discussion

The present results demonstrate a quite surprising and totally unexpected substantial reduction of the ipsilesional hand representation in M1, as assessed by ICMS, after unilateral section of the CS tract at cervical level. This observation appears robust since it was present in all of the three monkeys examined here. Indeed, the CS undecussated projection originating from the ipsilesional M1 and affected by the unilateral cervical lesion represents only 5–10% of the whole CS tract [38,40,41]. Moreover, the ICMS effects were assessed here only for the contralesional hand (Fig. 2) and thus the unilateral cervical lesion would impact only on the undecussated CS axons that cross the midline at cervical level, representing themselves only a small fraction (about 1/6) of the population of CS undecussated axons (as assessed elegantly by multiple tracing studies in monkeys subjected to cervical hemi-section at C3 level; [39]). In other words, based on these numbers (1/6 of 5–10% of CS axons), one would expect that the unilateral CS tract section would impact only marginally on the ICMS map in the ipsilesional hemisphere. In sharp contrast, the present data show a substantial reduction of the hand area projected on the surface of the ipsilesional hemisphere, amounting to 52%, 77% and 43% in Mk1, Mk2 and Mk3, respectively (Fig. 3), not far from the area reductions observed in the contralesional hemisphere (see [31]), amounting to 67%, 89% and 100% in Mk1, Mk2 and Mk3, respectively. In other words, the reduction of the hand area observed in the ipsilesional hemisphere was thus at least 50 times larger than expected, based on the very small contingent represented by the undecussated CS axons crossing the midline at cervical level.

It is important to stress that the reduction of the M1 hand area in the ipsilesional hemisphere as a result of unilateral cervical lesion in monkeys is an observation made at a specific time point, namely a few months post-lesion after the (incomplete) recovery of the affected hand had reached a plateau. The precise time course of such a reduction of the ipsilesional hand area during the few weeks post-lesion is unknown (in the absence of daily mapping) and one cannot exclude that the ipsilesional hand area was different than it appears after the recovery. Along this line, dynamic bi-hemispheric re-organization of motor networks during the recovery from hemi-paresis caused by corticospinal tract infarction has been observed [35]. Indeed, this study showed that the early recovery of the paretic hand was correlated to a predominant activity on the intact hemisphere but, in later phases of the recovery, the activity in the lesioned hemisphere increased. One cannot exclude such progressive changes of inter-hemispheric balance between the hand areas in the two M1 in our monkeys during the recovery period, leading to the final, fairly balanced size of hand areas between the two hemispheres after recovery reached its maximum.

### Comparison with previous work

The present observation of substantial plastic changes of somatotopic maps as a result of a peripheral lesion (at the level of spinal cord, or peripheral nerve lesion or amputation) is in line with an abundant literature on this topic. However, most previous studies in the monkey addressed this issue in the contralesional hemisphere with respect to the spinal cord lesion, either in the somatosensory cortex [29,30,39] or in the motor cortex [31]. In human subjects too, although it is relatively rare to have a cervical cord lesion restricted to one side, most studies aimed at assessing the cortical motor re-organization after spinal cord lesion were focused on the contralesional hemisphere [36,37]. In spinal cord injured patients, the cortical motor map changes consisted mainly of a displacement of the centre of gravity of cortical activity when using the paretic hand after partial recovery, towards a more posterior region [32], which was interpreted as a possible role played by the somatosensory cortex in recovery. In the monkey, as a result of unilateral cervical lesion, in the contralesional hemisphere [31] and in the ipsilesional one (present study), the ICMS data showed a reduction of the hand representation, but no evidence for a posterior shift of the hand representation was found. However, this discrepancy may also be explained by the difficulty to compare directly motor maps based on ICMS in the monkey and on cortical territories activated when performing movements in human. The present observation of a considerable re-organization of the motor map after unilateral cervical cord lesion in the ipsilesional hemisphere is, to our knowledge, an original observation in monkeys. Our finding can only be, to some extent, compared to previous observations in human subjects of functional reorganization in the ipsilesional hemisphere with respect to a cord damage due to lower limb amputation [43].

### Interpretation of the motor map changes in the ipsilesional hemisphere after unilateral cervical lesion

How to explain then a plastic change in M1 in the ipsilesional hemisphere, nearly as large as that in the contralesional hemisphere as a result of unilateral section of the CS tract? The reduction of the hand area in the contralesional hemisphere was correlated with anatomical changes such as a shrinkage of the soma of layer V pyramidal neurons, involving the 90% of the axotomized CS neurons [41]. In the ipsilesional hemisphere, we did not observe such shrinkage, as compared with intact animals [41], although this might have been difficult to detect since the unilateral cervical section would affect at most only 5–10% of the CS neurons, giving rise to the undecussated CS axons. In any case, the considerable plastic functional change observed for the hand representation in the ipsilesional hemisphere is not correlated with a major anatomical change (as far as the CS neurons are concerned), in contrast to the contralesional hemisphere. Consequently, the plastic change of motor map in the ipsilesional hemisphere most likely does not result from a direct impact of the axotomized CS tract. One may thus speculate that the size reduction of the hand area ipsilesionally is the result of more indirect (secondary) influences of the lesion. The present data support the notion that a reduction of the hand area in the contralesional hemisphere (which is expected) is accompanied by a nearly comparable reduction in the ipsilesional hemisphere. Although the M1 hand area is quantitatively less connected transcallosally than other body territories in M1 or other motor cortical areas such as SMA [44,45], one may still consider the possibility that the reduction of hand area in the contralesional hemisphere provokes a "secondary" plastic change in the ipsilesional hemisphere, via the callosal projection. The process of secondary change may also occur more indirectly via non-primary motor areas (premotor cortex, SMA), which are more densely connected via the corpus callosum, since lesions of the primary motor cortex induce modifications in the premotor cortex, for instance an extension of the hand area in the ventral premotor cortex [26]. Such "secondary" plastic change in the ipsilesional hemisphere may appear reminiscent to some extent of the transneuronal change observed in the brainstem and thalamus in adult monkeys subjected to long term dorsal rhizotomies [46]. However, a common mechanism is unlikely because, in the case of the rhizotomy there was an anterograde plastic change induced by a lesion, whereas here after cervical lesion the impact on the CS neurons is retrograde. Moreover, a transneuronal degeneration mechanism can be excluded because most axotomized CS neurons survived to the cervical lesion, although they shrank [41].

The extent of the ipsilesional reduction, nearly as large as in the contralesional hemisphere, suggests that such secondary adaptive plastic change may come about as a consequence of the re-balancing of activity in the two hand areas. A roughly balanced hand area in both hemispheres is perhaps more appropriate in the context of bimanual movements as well as in the context of the functional recovery of the ipsilesional hand. Indeed, after unilateral cervical lesion, the recovery of the ipsilesional hand strongly depends on the contralesional hemisphere [31] and not on the ipsilesional one (Fig. 6). If the hand area in the ipsilesional hemisphere had kept its original size after lesion, then there would be a bias in favor of the intact hand, which may be detrimental for mechanisms of recovery of the affected hand. Possibly, recovery may be more efficient if the cortical area responsible for it is not too much reduced in size as compared to its counterpart in the intact hemisphere. However, the hand area in the ipsilesional hemisphere should not be reduced too much either, because this may affect the performance of the contralesional hand. In the present study, as a result of unilateral section of the CS tract at cervical level, the reduction in size in the ipsilesional hemisphere did not affect the performance of the intact hand, at least as assessed by the modified Brinkman board test (Fig. 7) or the "drawer" task. One cannot exclude that a reduction of performance may appear for more challenging tasks, involving more complex synergies of the fingers. Along this line, one may speculate that the re-sizing in the ipsilesional hemisphere should be adjusted in order to reach an ideal compromise, favoring enough the recovery of the affected hand but preserving, as much as possible, the performance of the non-affected hand. The reduction of the hand area in the ipsilesional motor cortex may also be interpreted, at least in part, by postural adjustments as well as a facilitation of those movements not affected by the lesion, such as proximal movements and, but to a lesser extent, the wrist, taking place in the contralesional hemisphere. Such contralesional motor changes, for example comprising strategies of substitution recruited for the recovery, may secondarily induce changes in the ipsilesional hemisphere as well. Postural adjustments may also include the side of the body opposite to the unilateral cervical lesion, resulting in an increased engagement of more proximal muscles at the level of the wrist, elbow and shoulder in the ipsilesional hemisphere, at the expense of the hand representation.

Plastic changes of motor maps resulting from a cortical lesion have been shown to be dependent on the level of rehabilitative training [47,48]. It remains to be determined whether this would also be the case here in the contralesional hemisphere after cervical cord lesion and, if so, whether the same dependence on training would also be present in the ipsilesional hemisphere. In the present study, the monkeys did not undergo a particular and systematic rehabilitative training program, except for the standard behavioral tests they performed every day (essentially the modified Brinkman board test) to assess manual dexterity.

In the contralesional hemisphere, we demonstrated that rapid plastic changes of the hand motor map took place within the first few days post-lesion. During the 2 weeks after the unilateral cervical lesion, no ICMS digit sites were found [31]. Starting about 3 weeks after the lesion, ICMS digit sites progressively re-appeared, to form the stable, reduced hand area observed several months later. Unfortunately, such a repetitive ICMS investigation was not conducted in the ipsilesional hemisphere (because no change in the ipsilesional hemisphere was expected at the time of the experiments). Such a protocol is recommended for future experiments.

### Relationship between cortical plasticity and a possible role played for post-lesional recovery

Regarding the mechanisms of the incomplete recovery of the hand affected by the cervical lesion, the present study confirms the notion previously put forward [31] that only the contralesional hemisphere contributes to the recovered performance of the manual dexterity, as assessed by the precision grip task (Brinkman board). Indeed, reversible inactivation of the ipsilesional hemisphere did not modify the recovered manual dexterity score of the affected hand (Fig. 6). Nevertheless, we observed a change of the motor map in the ipsilesional hemisphere. It can thus be concluded that the presence of plastic changes in a certain brain region after a lesion does not necessarily mean that this region contributes significantly to the recovery. In other words, in the debate about whether the intact hemisphere plays a role in the recovery following a unilateral cortical lesion in patients (see e.g. [21-23]), a change of motor map area in the intact hemisphere as compared to normal human subjects should thus not be systematically interpreted as a contribution of the intact hemisphere to the recovery. Clearly, the strategy of reversible inactivation applicable to monkeys, as illustrated in the present study (Fig. 6), remains a better proof than just the observation of motor map changes for the actual involvement of a given brain region in mechanisms of recovery. Another conclusion of the present study is that there is no straightforward relationship between the size of the hand area in M1 (in the ipsilesional hemisphere) and the manual dexterity of the hand controlled mainly by this hemisphere. Indeed, the manual dexterity score was the same pre-lesion with a large hand area and post-lesion with a reduced hand area (Fig. 7). Furthermore, regarding the generation of force, the drawer task did not show a difference of performance pre- and post-lesion. This conclusion, valid for the motor tests used in the present study (Brinkman board and drawer tasks), may not be true for other types, of most likely more complex finger movements, as the activity in the ipsilateral motor cortex is related to the complexity of unimanual hand movements [16].

## Conclusion

As a result of unilateral section of the CS tract at cervical level, the hand representation in the contralesional motor cortex was as expected dramatically affected [31]. The present study demonstrates that a substantial post-lesional reduction of the hand representation also took place in the ipsilesional hemisphere, an original observation in the monkey. The considerable extent of the ipsilesional hand representation reduction cannot be explained by a direct effect of the lesion. Indeed, only a small number of transected CS axons originate from the ipsilesional hemisphere and could have contributed to the control of the hand opposite to the lesion by recrossing the midline below the lesion. We therefore propose that the paradoxical reduction of hand representation in the ipsilesional hemisphere is secondary to the changes taking place in the contralesional hemisphere, possibly corresponding to re-adjustments re-establishing a balance between the two hemispheres.

## Methods

### Overview of the experiments

The surgical procedures (anesthesia, physiological monitoring of the animal, implantation of chronic recording chamber above M1, post-operative care) were described in detail in previous reports from this laboratory [4,5,25,31,41,49]. The experiments were conducted in three young adult male (3–4 years old) Rhesus monkeys (Macaca mulatta), Mk1, Mk2 and Mk3, weighing around 4 kg, and subjected to a unilateral section of the CS tract at cervical level C7/C8. Control experiments to test the reproducibility of the intracortical microstimulation technique were conducted in a fourth, intact monkey (Mk4; Macaca fascicularis, weighing about 3 Kg). Mk1 and Mk2 are the same two animals included in the description of motor maps changes taking place in the contralesional hemisphere [31] and in the anatomical modifications in M1 resulting from the cervical lesion [41]. Mk3 underwent a comparable unilateral cervical lesion as Mk1 and Mk2, but was in addition treated during 4 weeks post-lesion with an antibody aimed at neutralizing the neurite growth inhibitor Nogo (see e.g. [50,51]). The antibody was delivered from an osmotic pump, placed in the back of the animal, using a small silastic tube positioned intrathecally 3–5 mm above the cervical lesion. The effect of the anti-Nogo treatment in Mk3 will be reported elsewhere. Mk1 and Mk2 were also implanted during 4 weeks with an osmotic pump, but delivering a control antibody. Surgical procedures and animal care were conducted in accordance with the Guide for the Care and Use of Laboratory Animals (ISBN 0-309-05377-3; 1996) and approved by local (Swiss) veterinary authorities.

### Assessment of manual dexterity

The manual dexterity of each hand was assessed in Mk1, Mk2 and Mk3 using our modified "Brinkman board" task, as described in detail earlier [25,31,52], testing the ability to grasp a food pellet using the opposition of the thumb and the index finger (precision grip). The "Brinkman board" is a Perspex board (10 cm × 20 cm) with 50 randomly distributed holes (15 mm long, 8 mm wide and 6 mm deep) containing each a pellet; 25 holes were oriented horizontally and 25 vertically. The task was performed daily (lasting for 15 to 20 minutes) for several months before and several months after the spinal cord lesion. All sessions were recorded on a video tape and one to two weekly sessions were analyzed quantitatively. An attempt was considered as successful when the monkey grasped a pellet and transported it to the mouth. The manual dexterity was quantitatively measured as the number of slots successfully retrieved within 45 seconds. In addition to the precision grip tested with the "Brinkman board" task, Mks 1–3 were also examined using the so-called "reach and grasp drawer task" [4-6,53-55]. Using one forelimb (unimanual "drawer" task), the monkey had to grasp the knob of a drawer, generate enough force to pull the drawer and, finally, grasp a reward placed inside a well dug in the drawer. By means of different sensors, it was possible to measure several time intervals, separating different epochs of the task. The left and the right forelimbs were tested separately, in series of 20 trials for each forelimb (once a week).

### Intracortical microstimulation experiments

The somatotopic organization in and around the hand area in M1 in both hemispheres was established based on daily ICMS sessions, as recently reported [31], using standard parameters of stimulation: 35 ms duration trains of 12 electric monophasic pulses (0.2 ms) presented once every 2 seconds, through a tungsten microelectrode (FHC, Maine, USA) with an impedance of 0.1 – 0.6 MOhms and a tip of about 20–30 μm. Electrode penetrations were oriented nearly perpendicular to the cortical surface, and ICMS was applied at 1 mm steps along the entire track, starting 2 mm below the dura and down to a depth of usually 8–10 mm, sometimes even deeper when the penetration went all the way down to the rostral bank of the central sulcus. On surface ICMS maps (see Figs. 3 and 4), each electrode penetration was represented by a single point corresponding to its position of entry in the brain. ICMS investigation was focused on the hand area with determination of the body territories represented a few mm around the representation of the fingers. The term "hand area" thus refers to the ensemble of ICMS sites in the motor cortex eliciting movements of the fingers observed on the contralateral hand.

The intact monkey Mk4 was included in the present study with the specific aim of assessing the variability of the ICMS method. The hand area of Mk4 was extensively mapped using the ICMS technique as described above. In the left hemisphere, six electrode penetrations selected among different body territories ("face", "wrist" and "digits") were repeated at two time points separated by a time interval ranging between 12 and 140 days.

### Reversible inactivation experiments

Mks1-3 were subjected to a unilateral section of the CS tract at C7/C8 level, as described in detail recently [31,41]. Three to five months post-lesion, a time at which the incomplete recovery process had reached a plateau, sessions of reversible inactivation of M1 in either hemisphere using muscimol were conducted, as previously described in detail [25,31,54]. Two to four ICMS penetration sites were chosen in the pre-lesion hand area of M1 in one or the other hemisphere (see syringes in Fig. 3) and the GABA-agonist muscimol (1 μg in 1 μl saline) was infused at two depths along each penetration (separated from each other by 2–3 mm). The infusion of muscimol was performed at ICMS sites at which finger movements were elicited at low threshold and were separated from each other in order to cover the entire hand area, based on previous experiments [25,54]. In Mk1, three and five months post-lesion, two muscimol inactivation sessions were conducted on the left (ipsilesional) hemisphere, 7 weeks apart from each other. In each inactivation session, the total volume of muscimol injected was 18 μl, along three penetrations (Fig. 3, top left panel). In Mk2, only one muscimol inactivation session was conducted on the ipsilesional hemisphere five months post-lesion, in which a total volume of 12 μl of muscimol was infused along two penetrations (Fig. 3, middle left panel). Finally, in Mk3, one reversible inactivation session took place 4 months after the lesion, in which a total volume of 24 μl of muscimol was infused along four penetrations (Fig. 3, bottom left panel). For comparison, in the three monkeys, inactivation sessions were also conducted for the contralesional hemisphere in which muscimol infusion sites were also selected based on ICMS data (not shown; see however [31] for Mk1 and Mk2). The efficacy of such reversible inactivation protocol has been demonstrated previously, together with control experiments in which only saline was injected [54].

## List of abbreviations

AR = arcuate sulcus

CE = central sulcus

CMA = cingulate motor area

CS = corticospinal

D = digit

E = elbow

F = face

ICMS = intracortical microstimulation

LH = left hemisphere

M1 = primary motor cortex

Mk = monkey

PM = premotor cortex

RH = right hemisphere

S = shoulder

SMA = supplementary motor area

W = wrist

## Authors' contributions

EMR designed the study, helped with the reversible inactivation experiments, to the analysis of the behavioral and ICMS data and drafted the manuscript. ES and TW designed the study and carried out the behavioral, reversible inactivation, ICMS experiments and analyzed the corresponding data, including the histological assessment of the lesion. JB designed the study and performed the cervical lesions. ABS and AFW carried out the experiments related to the reproducibility of the ICMS methods. All authors read and approved the final manuscript.

## Figures and Tables

**Table 1:** ICMS effects in the ipsilesional hemisphere. For each lesioned monkey, the total number of ICMS sites tested is given in the rightmost column, separately for the ICMS sessions pre- and post-lesion. These ICMS sites tested were then distributed in three groups, depending on whether no effect was observed ("non-microexcitable" sites) or elicited movements of the hand ("digit" sites) or movements of other body territories ("other territories"), such activation of wrist, elbow, shoulder or face muscles. Between parentheses, the number of ICMS sites in each group is given in %. For each line, the sum of the three groups is 100%.

	Nb. of ICMS "digit" sites	Nb. of ICMS "other territories" sites	Nb. of "non-microexcitable" sites	Total
**Mk1**				
Pre-lesion	79	77	57	213
%	(37.1)	(36.2)	(26.7)	(100)
				
Post-lesion	39	78	82	199
%	(19.6)	(39.2)	(41.2)	(100)
				
				
**Mk2**				
Pre-lesion	81	72	139	292
%	(27.7)	(24.7)	(47.6)	(100)
				
Post-lesion	29	70	158	257
%	(11.3)	(27.2)	(61.5)	(100)
				
				
**Mk3**				
Pre-lesion	53	68	150	271
%	(19.6)	(25.1)	(55.3)	(100)
				
Post-lesion	20	37	207	264
	(7.6)	(14.0)	(78.4)	(100)
